# Hfq Regulates Efflux Pump Expression and Purine Metabolic Pathway to Increase Trimethoprim Resistance in *Aeromonas veronii*

**DOI:** 10.3389/fmicb.2021.742114

**Published:** 2021-11-24

**Authors:** Dan Wang, Hong Li, Xiang Ma, Yanqiong Tang, Hongqian Tang, Dongyi Huang, Min Lin, Zhu Liu

**Affiliations:** ^1^College of Life Sciences, Hainan University, Haikou, China; ^2^College of Tropical Crops Hainan University, Haikou, China; ^3^Chinese Academy of Agricultural Science, Beijing, China

**Keywords:** *Aeromonas veronii*, Hfq, trimethoprim, antibiotic resistance, *acrA*/*acrB*, purine pathway

## Abstract

*Aeromonas veronii (A. veronii*) is a zoonotic pathogen. It causes clinically a variety of diseases such as dysentery, bacteremia, and meningitis, and brings huge losses to aquaculture. *A. veronii* has been documented as a multiple antibiotic resistant bacterium. Hfq (host factor for RNA bacteriophage Qβ replication) participates in the regulations of the virulence, adhesion, and nitrogen fixation, effecting on the growth, metabolism synthesis and stress resistance in bacteria. The deletion of *hfq* gene in *A. veronii* showed more sensitivity to trimethoprim, accompanying by the upregulations of purine metabolic genes and downregulations of efflux pump genes by transcriptomic data analysis. Coherently, the complementation of efflux pump-related genes *acrA* and *acrB* recovered the trimethoprim resistance in Δ*hfq*. Besides, the accumulations of adenosine and guanosine were increased in Δ*hfq* in metabonomic data. The strain Δ*hfq* conferred more sensitive to trimethoprim after appending 1 mM guanosine to M9 medium, while wild type was not altered. These results demonstrated that Hfq mediated trimethoprim resistance by elevating efflux pump expression and degrading adenosine, and guanosine metabolites. Collectively, Hfq is a potential target to tackle trimethoprim resistance in *A. veronii* infection.

## Introduction

*Aeromonas veronii (A. veronii)* is a rod-shaped gram-negative pathogen found in diseased grass fish, tilapia, and turtles. It can cause huge losses in the aquaculture industry but also infect humans (Liu et al., 2015; Wang et al., 2019). *A. veronii* performs multiple drug resistance to antibiotics such as ampicillin, kanamycin, streptomycin, and gentamycin, resulting in the increased risk of human diseases and the greater losses to the fishery (Liu et al., 2017; Wang et al., 2019; Zhang et al., 2019). Hfq is a relatively common molecular chaperone that interacts with small RNAs to mediate the binding of small RNA to mRNA and assists in the post-transcriptional regulation of bacterial genes. Hfq participates in several regulatory pathways as a global regulator (Kakoschke et al., 2016). Deletion of *hfq* reduces tolerance to harsh environments in Escherichia *coli, Salmonella enterica*, and *Vibrio hollisae* (Yamada et al., 2010; Hayashi-Nishino et al., 2012; Azam and Vanderpool, 2018). Hfq is involved in regulating the virulence of *Aeromonas hydrophila*, the adhesion of *Vibrio alginolyticus*, and the nitrogen fixation efficiency and plant interactions of *Pseudomonas stutzeri* (Hayashi-Nishino et al., 2012; Kakoschke et al., 2016; Azam and Vanderpool, 2018; Santiago-Frangos and Woodson, 2018).

Albeit there are few studies on Hfq-related drug resistance, Hfq is documented to affect multidrug resistance of *E. coli* (Yamada et al., 2010). In this study, the *hfq* knockout strain (Δ*hfq*) of *A. veronii* conferred more sensitive to trimethoprim. Trimethoprim is a broad-spectrum antibacterial that inhibits the activity of dihydrofolate reductase and the synthesis of tetrahydrofolate (Stepanek et al., 2016). Tetrahydrofolate is a one-carbon unit donor that provides the raw materials needed to synthesize purine nucleotides and thymidine nucleotides *in vivo*. Tetrahydrofolate is concerned with the regulation of purine metabolism and is closely related to bacterial nucleic acid synthesis, energy metabolism, ion transport and signal transduction (Jinnah et al., 2013; Vazquez-Salazar et al., 2018).

In addition, the absence of *hfq* affects the expression of the efflux pump gene *acrAB* (Spaniol et al., 2015). AcrB belongs to the resistance-nodulation-cell division superfamily (RND). The substrates for the AcrAB-TolC efflux pump include a variety of antibiotics, detergents, bactericides, fuels, and free fatty acids (Li et al., 2015; Phetsang et al., 2016). The efflux pump can excrete trimethoprim, affecting the antibacterial effect of trimethoprim (Köhler et al., 1996; Podnecky et al., 2013). The efflux pump AcrAB accounts for a large proportion of the mechanisms of bacterial antibiotic resistance (Elsby et al., 2017; Zwama et al., 2018). Therefore, it is hypothesized that the sensitivity of *A. veronii* to trimethoprim may be related to the expression of the efflux pump. In summary, Hfq indirectly affects the sensitivity of trimethoprim by affecting purine metabolism and efflux pumping; these effects are important for further understanding of the molecular mechanisms of multidrug resistance.

## Materials and Methods

### Strains and Culture

The strain information was listed in Table 1. The derivative *A. veronii* strains included wild-type, Δ*hfq*, Δ*hfq:hfq*, and Δ*hfq:acrAB.* The strains were cultured in M9 minimal medium (M9) at 30^°^C, 150 r/min, supplemented with 50 μg/mL ampicillin. Δ*hfq* represents the *hfq* knockout strain (Zhang et al., 2019). Δ*hfq:acrAB* overexpresses the *acrAB* gene in the *hfq* knockout strain. The strain *E. coli* WM 3064 was applied to assist in the introduction of the plasmids into *A. veronii* by tri-parent conjugation (Simon et al., 1983; Ferrieres et al., 2010). For the culture of strain WM3064, 0.3 mM diaminopimelic acid was supplemented in LB at 37^°^C.

**TABLE 1:** Strains and plasmids used in this paper.

Strains or plasmids	Traits	Sources
*E. coli* WM3064	Gene cloning strain	Liu et al., 2016
*Aeromonas veronii*	Wild type strain	Liu et al., 2016
*Δhfq*	*hfq* deletion mutant	Zhang et al., 2019
*Δhfq::hfq*	*hfq* complement strain	Zhang et al., 2019
*Δhfq::acrAB*	acrAB overexpression in hfqmutant strain	This paper
pBBR1MCS-2	Gene cloning vector	Zhang et al., 2019
pBBR *acrAB*	acrA/B overexpression vector	This paper

### Vector and Primers

The vector and primers were listed in Table 2. For the construction of the expression vector of the efflux pump-associated gene *acrAB*, the *acrA* and *acrB* genes were inserted into the plasmid pBBR1MCS-2, wherein the enzyme cleavage sites were *Sal*I and *Eco*RI. The upstream and downstream primers required for the construction were *acrA* F 5′-ACGCGTCGACTTGGTATCGGCTGGGGATTG-3′ and *acrB* 5′-CCGGAATTCATGAGCGTCGGGAGAG-3′.

**TABLE 2:** Primers used in this paper.

Names of primers	Sequences (5′-3′)	Usage
WP_041202667.1-F	ATGGTCGCAGAGCTTGTC	Strain validation
WP_041202667.1-R	CAGCACAATAGAACACCAGAC	Strain validation
*acrA Sal*I F	ACGCGTCGACTTGGTATCG GCTGGGGATTG	*acrAB* vector construction
*acrB Eco*RI R	CCGGAATTCATGAGCGTCGGGAGAG	*acrAB* vector construction
pBBR1MCS-2 F	GGCACCCCAGGCTTTACACT	Complement plasmid validation
pBBR1MCS-2 R	GATGTGCTGCAAGGCGATTAAG	Complement plasmid validation

### Minimum Inhibitory Concentration Test

Antibiotics were added to sterile 96-well plates at final concentrations of 64, 32, 16, 8, 4, 2, 1, 0.5, 0.25, and 0.125 μg/mL (Andrews, 2001). Then, 10^6^ CFU broth was added to each well to a final volume of 200 μL. The 96-well plate was sealed with parafilm and cultured at 30^°^C with shaking at 150 r/min for 24 h. The experiment was repeated for 3 times.

### Transcriptomic Analysis

The wild type and Δ*hfq* strain were cultured in M9 medium containing 50 μg/mL ampicillin, cultured at 30^°^C and 150 r/min for 20 h, centrifuged to remove the culture medium, and washed with sterile PBS for transcriptomic analysis. The sequencing was carried out by BGI (Beijing Genomics Institution). The cells were collected and lysed, and the sample RNA was extracted with phenol-chloroform. The concentration and quality of the RNA samples were tested with the Agilent 2100. DNase I was used to remove double-stranded DNA, and a Ribo-Zero Magnetic Kit was used to remove ribosome RNA. Reverse transcription was performed with random primers and first strand cDNA as a template to synthesize the second strand. The linker sequence was attached to the 3′ end of the cDNA fragment. The cDNA sequence was amplified with a primer cocktail, and the purified product was sequenced on a HiSeq Xten (Illumina, San Diego, CA, United States) platform. The sequencing depth was chain-specific sequencing for 2 Gb of clean data. HISAT was attempted for genome assembly, potential coding sequence analysis and new transcript identification that may be present. The transcriptional differences between wild-type and *hfq* knockout were analyzed by Bowtie 2, and FPKM was used to normalize gene expression levels. Each gene expression was calculated using the Benjamini-Hochberg false discovery rate (FDR). The differential transcripts were tested for log-fold change, and the *p* value was corrected with FDR < 0.001. The differential genes were analyzed using GO classification, and disparity expression in the pathway was compared with the entire genomic background using hypergeometric analysis *p* ≤ 0.05 was a differential metabolic pathway. GEO accession number was GSE120603, and the URL of accession website was displayed as https://submit.ncbi.nlm.nih.gov/subs/sra/SUB6133286. The DESeq. 2 packages in R were applied to estimate the fold changes and perform other analysis. *A. veronii* TH0426 genome (Genomic Sequence: NZ_CP012504.1) was referenced for transcriptome analysis (Kang et al., 2016).

### Metabolomics Analysis

The non-target metabolomic and lipidomic detection platform (UHPLC-QTOF-MS) was applied to metabolomics for the detection of *A. veronii* samples. UHPLC-QTOF-MS included Ultra-Performance Liquid Chromatography 1290UHPLC (Agilent), ACQUITY UPLC BEH Amide column 1.7 μm, 2.1 × 100 mm (Waters) and High-Resolution Mass Spectrometry Triple TOF 6600 (AB Sciex). The original mass spectrum was converted to the mzXML format using Proteo Wizard software, and the peaks were identified using the R Programming Language package (Version 3.2) and self-built secondary mass spectrometry data. URL of accession website was displayed as www.ebi.ac.uk/metabolights/MTBLS1411.

### Statistical Analysis

Statistical data were analyzed using the statistical Package for the Social Science (SPSS) version 20.0 (SPSS, Chicago, IL, United States) and GraphPad Prism version 8.0 (GraphPad, San Diego, CA, United States). The results are presented as the mean values of three independent experiments with standard deviation using one-way analysis of variance. *p* < 0.05 or 0.01 were represented as significant or extremely significant, respectively.

## Results

### Hfq Deletion Reduces Multiple Resistance to Antibiotics Including Trimethoprim

**FIGURE 1:**
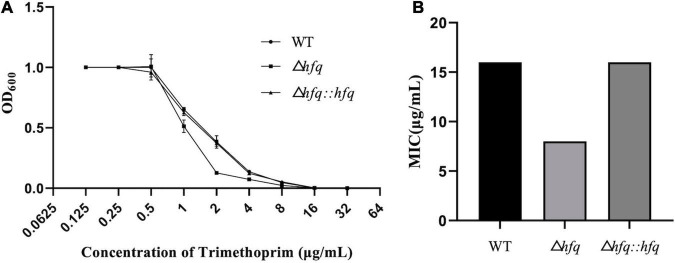
Growth and Minimum inhibitory concentration (MIC) of trimethoprim. **(A)** The growth of three *A. veronii* strain: wildtype, Δ*hfq*, and Δ*hfq:hfq*. **(B)** The MIC of trimethoprim of wildtype, Δ*hfq*, and Δ*hfq:hfq.*

According to previous studies, *A. veronii* were resistant to gentamycin, kanamycin, streptomycin, and were sensitive to chloramphenicol, ciprofloxacin (Liu et al., 2018). Trimethoprim, as an antibiotic that inhibits folic acid metabolism, has a strong inhibitory effect on a variety of bacteria. Resistance to trimethoprim was found to be altered in the absence of hfq (Figure 1A). The mutant Δ*hfq* was more sensitive to trimethoprim than wild type, which exhibited with a minimum inhibitory concentration (MIC) of 8 μg/mL in contrast to 16 μg/mL of wild type. The complemented strain attenuated the sensitivity of Δ*hfq* to trimethoprim, and the MIC was the same as that of wild type (Figure 1B).

### Upregulation of Purine Metabolic Gene Expression and Downregulation of Efflux Pump-Related Genes in Δ*hfq* Strain

**FIGURE 2:**
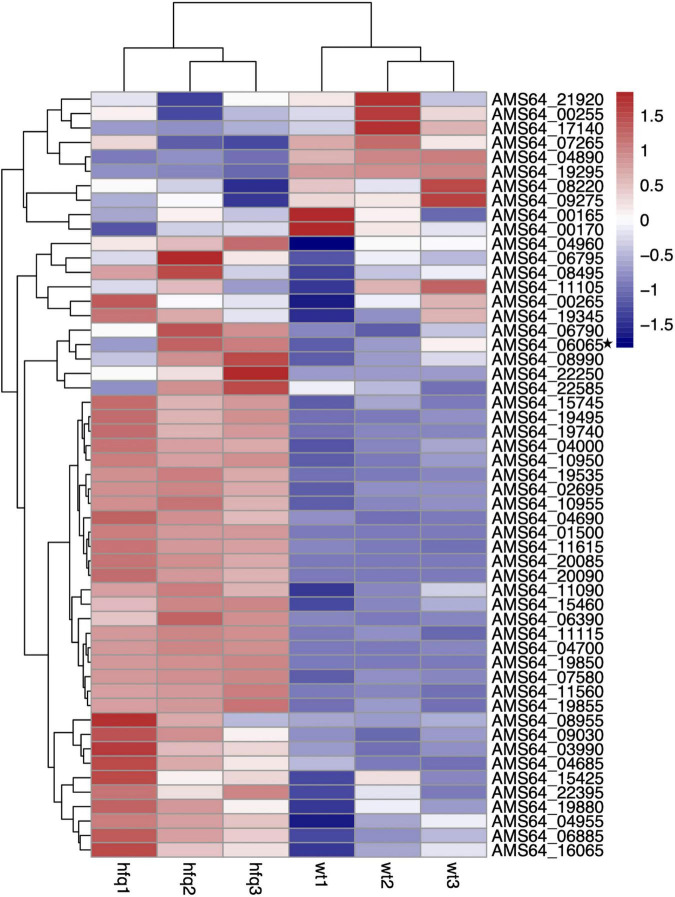
Transcriptomic data of *Aeromonas veronii*. Heat map of expression levels of genes involved in purine metabolism and efflux pump synthesis of wild-type and Δ*hfq* strains.

**FIGURE 3:**
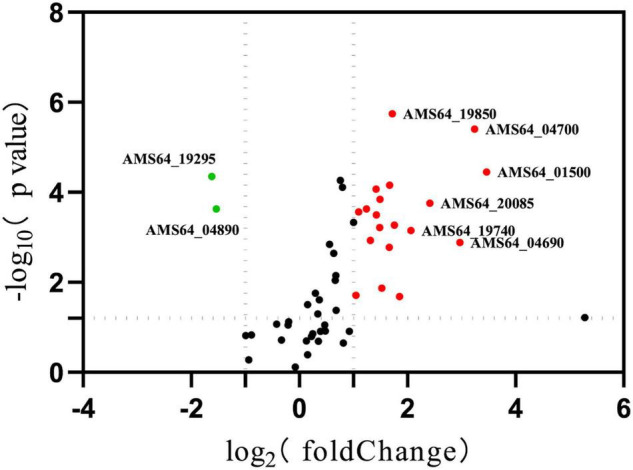
Expression of genes related to purine metabolism. Green represents down-regulated genes with significant differences. Red indicates up-regulated genes with a significant difference. *represents *p* < 0.05.

To understand the changes of drug resistance in Δ*hfq* strains, transcriptome sequencing was used to compare metabolic pathways with significant variations in expression levels and to analyze their relationship with trimethoprim resistance. The clustering analysis revealed that many genes related to purine metabolism and efflux pump synthesis were expressed differently (Figure 2). Although the direct target of trimethoprim was dihydrofolate reductase, the transcriptions of dihydrofolate reductase were not significantly different between Δ*hfq* and wild type (Figure 2 marked with star). However, the expression of 53 genes was affected in purine metabolism, which functioned as the downstream of folate metabolism (Figure 3). There were 21 genes marked with red were up-regulated which led to purine accumulation. Two genes marked with green, as purine consuming enzymes including xanthine nucleic acid transferase and hypoxanthine nucleic acid transferase, were significantly down-regulated.

### Metabolomics Analysis Displays That Δ*hfq* Enhances Purine Metabolism

**FIGURE 4:**
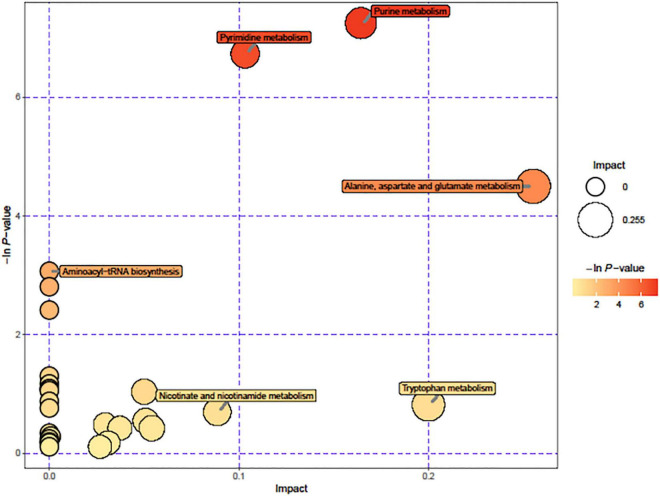
Pathways affected by *hfq* knockout. The circle diameter indicates the degree of influence of the pathway, and the –ln (*p* value) and color indicate the significant difference in the number of genes regulated by the pathway for the entire genomic background.

**FIGURE 5:**
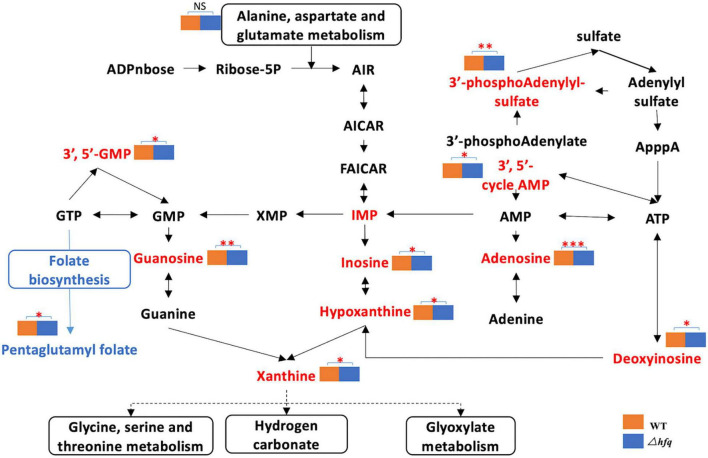
Metabolites of the purine metabolic pathway of *Aeromonas veronii*. The red font indicates the increased metabolites, and the blue color indicates the decreased metabolites. The metabolic pathway is framed by a solid line. * represents the difference between Δ*hfq* and wild-type metabolites. * represents *p* < 0.05, ^**^ represents *p* < 0.005, and ^***^ represents *p* < 0.001.

Due to the significant changes of transcription in metabolic pathways, variant metabolites of these pathways have been hypothesized to be responsible for trimethoprim resistance. The differential metabolites of wild-type and Δ*hfq* were screened, analyzed, and classified into metabolic pathways (Figure 4). Purine metabolism, pyrimidine metabolism, and alanine, aspartate, and glutamic acid metabolism were greatly affected by the deletion of *hfq*, of which the effects on purine metabolism and pyrimidine metabolism were prominent. The deletion of Hfq incurred a significant increase of purine metabolites including adenosine, guanosine, and xanthine (Figure 5).

### The Accumulation of Purine Metabolites Enhances the Sensitivity of Δ*hfq* to Trimethoprim

**FIGURE 6:**
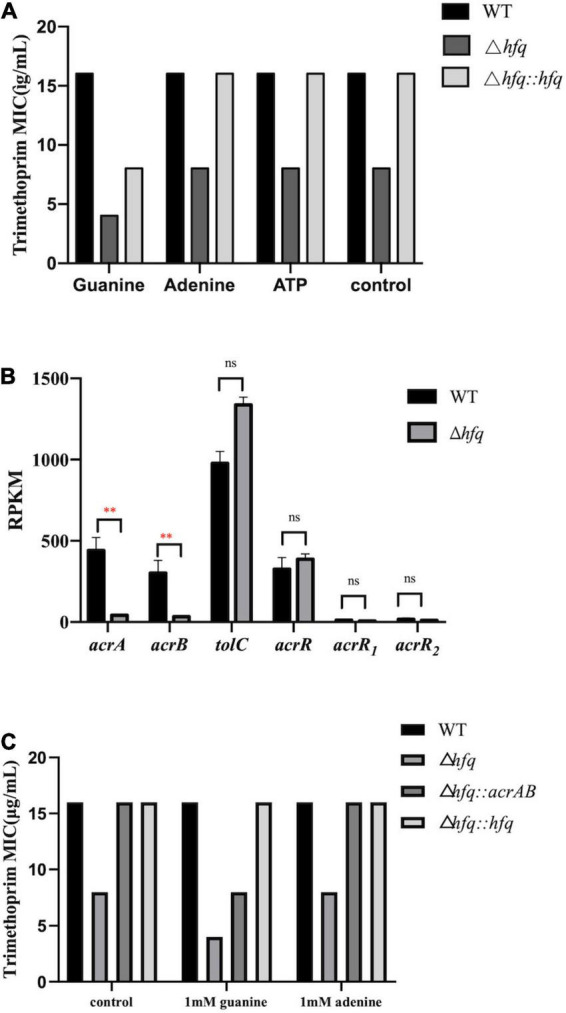
MIC of trimethoprim under conditions of exogenous purine metabolites and overexpressed AcrAB. **(A)** The concentrations of exogenous products are 1 mM for adenosine, guanosine, and adenosine triphosphate, and ATP. Equal volume of ddH_2_O was added as a control group **(B)**. Expression of key genes in the efflux pump. The rpkm of three *acrR* copies was presented separately. **(C)** MIC of trimethoprim to *Aeromonas veronii* overexpressing *acrAB*. * represents *p* < 0.05, ^**^ represents *p* < 0.01, and ns represents no significant difference.

The purine metabolites, such as guanosine and adenosine, were significantly increased in Δ*hfq* compared with wild type (Figure 3). To understand whether the accumulation of metabolites changed the trimethoprim sensitivity of *hfq* knockout, the downstream products such as 1 mM adenine, 1 mM guanine, and 1 mM ATP were added to the M9 medium to evaluate the MIC separately (Yang et al., 2019). The MIC of wild type was not altered when supplemented with 1 mM guanosine (Figure 6A), while that of Δ*hfq* was decreased. There had little evident changes both in wild type and Δ*hfq* after appending with 1 mM adenine or ATP. The above results suggested that the additional guanine enhanced the sensitivity of Δ*hfq* to trimethoprim.

### Overexpression of *acrAB* Enhances the Tolerance to Trimethoprim

AcrAB-TolC is capable of actively transporting antibiotics (Li et al., 2015), and trimethoprim can be transported outside the cell membrane by an efflux pump of *P. aeruginosa*. According to the transcriptomic data, the mRNA levels of *acrA* and *acrB* in Δ*hfq* were significantly reduced (by 9.30-fold and 9.34-fold) compared with wild type, but those of three copies of transcriptional repressor *acrR*, and that of component *tolC* (by 1.35-fold) were transcribed consistently (Figure 6B). The *acrAB* overexpression vector was constructed and transferred into the Δ*hfq* strain. The overexpression strain Δ*hfq:acrAB* showed an increased MIC and enhanced tolerance compared with Δ*hfq*. Overexpression of *acrAB* reversed the loss of *hfq*, resulting in inefficient discharge of trimethoprim (Figure 6C).

## Discussion

As a small chaperone protein, Hfq regulates gene expression by binding to sRNA and mRNA in response to external stress and environmental changes. Previous studies revealed that Hfq acts on a variety of membrane-associated protein genes, affecting bacterial growth, cell membrane formation, virulence, drug resistance, stress tolerance, and retention of retained bacteria (Hayashi-Nishino et al., 2012; Zhang et al., 2019).

Aeromonas *veronii* is highly resistant to ampicillin, kanamycin, gentamicin, streptomycin, and spectinomycin (Liu et al., 2016; Zhang et al., 2019). Previously the MIC of *hfq* knockout strain is significantly lower than that of wild type under the treatment of antibiotics (Zhang et al., 2019). As the substrate of nucleotide, the related genes and products of purine pathway showed significant differences in Hfq mutant strain (Figures 3, 5). But in fact, the productions of purines and nucleotides are affected by one carbon unit carrier tetrahydrofolate, and the latter is controlled by dihydrofolate reductase in turn (Paulsen et al., 2013). Since antibiotic trimethoprim targets dihydrofolate reductase specifically (Darrell et al., 1968; Bourne, 2014; Toulouse et al., 2020), trimethoprim is treated for Hfq knockout instead of other antibiotics. There are many mechanisms for resistance, of which efflux pump is important for multidrug resistance in bacteria (Elsby et al., 2017). The active transport function of the efflux pump is one of the main reasons for the decreased resistance to antibiotics (Abdali et al., 2017). The efflux pump AcrAB-TolC is an RND-type efflux pump that transports antibiotics through the inner membrane, periplasmic cavity, and outer membrane to the outside of the bacteria (Wang et al., 2017; Shi et al., 2019). The downregulations of the efflux pump-related genes *acrA* and *acrB* interfere with the assembly of the efflux pump, which reduce the ability of the efflux pump to bind and transport antibiotics and increase the sensitivity of the bacteria to trimethoprim (Cunrath et al., 2019). As a negative regulator of *acrAB*, the transcription level of *acrR* maintained a consistent in Δ*hfq*, indicating that *acrAB* was regulated independently by Hfq rather than AcrR.

The enzymes of purine metabolism were enhanced in Δ*hfq* strain, companying with the augmented productions of intermediate metabolites guanosine and adenosine. However, the quantities of downstream metabolites including glutamine, serine, threonine and glyoxylate were not significantly altered (Figure 3). In this study, metabolomics data showed that the deletion of *hfq* gene influenced on the basal metabolic pathways such as bacterial energy metabolism, hydrazine, and pyrimidine anabolism (Figures 4, 5).

Our experimental results demonstrated that Hfq affected the sensitivity of *A. veronii* to trimethoprim through different pathways. The downregulation of efflux pump system genes reduced the assembly of the efflux pump complex and decreased the ability of the cell to export trimethoprim. The transcriptional upregulation of many genes in purine metabolic pathway recruited the accumulation of metabolites, making *A. veronii* more sensitive to trimethoprim.

## Data Availability Statement

The datasets presented in this study can be found in online repositories. The names of the repository/repositories and accession number(s) can be found in the article/supplementary material.

## Author Contributions

ZL, XM, ML, DH, and DW contributed the conception and design of the study. DW, HL, YT, and HT performed the statistical analysis. DW and ZL drafted the manuscript. All authors contributed to manuscript revision, read, and approved the submitted version.

## Conflict of Interest

The authors declare that the research was conducted in the absence of any commercial or financial relationships that could be construed as a potential conflict of interest.

## Publisher’s Note

All claims expressed in this article are solely those of the authors and do not necessarily represent those of their affiliated organizations, or those of the publisher, the editors and the reviewers. Any product that may be evaluated in this article, or claim that may be made by its manufacturer, is not guaranteed or endorsed by the publisher.
